# Finding Effective and Efficient Ways to Integrate Research Advances Into the Clinical Suicide Risk Assessment Interview

**DOI:** 10.3389/fpsyt.2022.846244

**Published:** 2022-02-25

**Authors:** M. David Rudd, Craig J. Bryan

**Affiliations:** ^1^Department of Psychology, University of Memphis, Memphis, TN, United States; ^2^Department of Psychiatry and Behavioral Science, The Ohio State University Wexner Medical Center, Columbus, OH, United States

**Keywords:** suicide risk assessment, clinical interview, recent advances, efficiency, effectiveness

## Abstract

Research in clinical suicidology continues to rapidly expand, much of it with implications for day-to-day clinical practice. Clinicians routinely wrestle with how best to integrate recent advances into practice and how to do so in efficient and effective fashion. This article identifies five critical domains of recent research findings and offers examples of simple questions that can easily be integrated into a clinician's existing suicide risk assessment interview and related protocol helping inform the risk formulation process.

## Introduction

The last several decades have witnessed a sharp, positive trajectory in suicide-related research, much of it with direct and important implications for day-to-day clinical practice (1). In particular, five identifiable domains of research are of importance for practicing clinicians, including recent work demonstrating: (a) the limited predictive value of traditional suicide risk scales (e.g., the Columbia Suicide Severity Rating Scale, C-SSRS) in real-world healthcare settings (2, 3), (b) the temporal dynamics and natural variability of suicidal ideation and motivation to die across clinical and non-clinical populations [e.g., (4–7)], (c) the importance of assessing constructs other than suicidal ideation that are convincingly linked to enduring risk or chronic vulnerability for suicide [e.g., (8–10)], (d) the importance of understanding and assessing the potential for poor individual adherence and cooperation with clinical care (8), and (e) the elegant utility of patients' expressed wish to live and wish to die, coupled with reasons for living and reasons for dying (11–13). Regardless of the clinician's preferred theoretical perspective or approach, findings across all five of these domains can easily and efficiently be integrated into the suicide risk assessment interview, with straightforward questions that carry very little time-burden for the clinician and/or patient, while potentially capturing data essential to efforts to accurately understand, assess and respond to suicide risk.

Clinicians routinely struggle with how best to integrate a range of formal suicide risk assessment tools into day-to-day practice. Although suicide risk assessment screening tools are almost uniformly recommended, their limited predictive value is readily recognized and acknowledged [e.g., (14–16)]. We are not arguing against the use of formal suicide risk screening and assessment tools, as they serve an important and essential role in the overall risk assessment process. However, the problem of poor predictive value is compounded by clinicians having to juggle significant time demands, coupled with the challenge of finding effective ways to create a compassionate and caring assessment environment that will increase the likelihood of accurate self-report, increase self-disclosure during the clinical interview, and help develop a strong therapeutic alliance (17). There are a broad range of reasons why patients might be hesitant to accurately self-disclose on both assessment instruments and during the clinical interview, such as shame, the need for control/autonomy, limited emotional self-awareness/understanding, and disruption created by current symptoms [e.g., (18)]. All too often, the net result is that clinicians might overlook that many of these empirically supported constructs can be integrated into the clinical interview in simple and straightforward fashion, adding only a few minutes to the clinical interview.

Below are a few suggestions on how to incorporate some of these recent research advances into a standard clinical suicide risk assessment interview in a brief, but targeted manner. These suggestions are by no means intended to represent the entirety of a comprehensive suicide risk assessment [e.g., (15, 19)]; rather, they are only examples of simple strategies that can be used to supplement a clinician's existing suicide risk assessment interview and clinical decision-making process. Critical risk factors and domains assessed by existing tools, instruments and approaches are essential to an effective and comprehensive assessment of suicide risk (16); the material presented here should simply be viewed as supplemental in nature.

## Judicious Use of Suicide Risk Screening Tools

Suicide risk screening and assessment with a standardized instrument or scale is a recommended standard of care element in outpatient mental health, inpatient psychiatric, and emergency department settings (National Action Alliance for Suicide Prevention, 2018). Screening and assessment for suicidal ideation and behaviors in particular are recommended during the first clinical encounter (e.g., intake) and regularly during subsequent contacts. Screening tools and standard assessment instruments are only a starting point and one piece of information in a comprehensive suicide risk assessment process. Clinical decisions should not be made based solely on the scores obtained from these instruments, however, as considerable evidence shows that suicide risk screening tools have very poor accuracy and predictive value (2, 3). The accuracy of standardized instruments is reduced in part by the unwillingness (or even inability) of some individuals to reveal risk through direct and specific questioning (15), along with the observed temporal dynamics of suicidal thinking (4), a phenomenon that existing assessment tools are yet to meaningfully capture and measure. Clinical decisions should instead be made based on the integration of multiple data points from multiple sources (e.g., behavioral observations), which can help contextualize these scores, with the clinical interview arguably at the nexus. Expanding the clinician's available assessment toolkit with targeted clinical interview questions based on recent research advances will hopefully generate additional data to help inform and improve risk formulation and related clinical decision making.

## The Dynamic Nature of Suicidal Ideation

Standardized suicide risk instruments are ill-suited to assess the natural temporal dynamics of suicidal thinking. Suicidal ideation and motivation to die ebbs and flows, sometimes very rapidly. If the patient denies active suicidal thinking, the clinician should consider and explore the possibility of cycling in suicidal thinking and motivation to die, which has been linked with increased risk for suicidal behavior (5, 6, 20). More specifically, some individuals may accurately deny active suicidal thoughts and related intent to die, but also experience very rapid onset of specific suicidal thoughts and strong motivation to die, often with little forewarning. Recognizing such cycling is important to an accurate understanding of individual suicide risk. Potential shifts and cycling in suicidal thinking and desire to die for some individuals can be captured with a few simple questions:

Some people find that their suicidal thoughts and desire to die come and go, changing rapidly from minute to minute, hour to hour, or day to day? Does this describe you? Can you describe any pattern(s) you've noticed in your suicidal thinking? What is an average day or week like for you?

Since you don't report any active suicidal thoughts, if agreeable, let's focus on the last time you thought about suicide and felt motivated to die. When was the last time you thought about suicide? Let's focus on that episode to see if there's any identifiable pattern to how your thoughts about suicide and motivation to die come and go.

## Assessing Constructs Other Than Suicidal Ideation

Recent findings regarding the limited predictive value of traditional suicide risk assessment tools reinforces the need to assess other markers of enduring suicide risk that are strongly correlated with the emergence of suicidal behavior but are distinct from suicidal ideation and planning. For example, perceived burdensomeness (9), acquired capability (10), and identity-based hopelessness (21) have garnered considerable empirical evidence as useful indicators of heightened risk states. As with efforts to understand temporal dynamics, these constructs can be assessed with simple, straightforward questions. They also have the potential advantage of being indirect indicators of risk and create an opportunity to identify significant risk even when direct questions prove ineffective or active suicidal thoughts are denied. Similarly, entities such as the Royal Australian and New Zealand College of Psychiatrists (22) have emphasized the importance of a comprehensive approach to suicide risk assessment that considers variables identified by the individual as uniquely contributing to their suicide risk (e.g., homelessness, bullying, rejection). As summarized at the end of this article, these unique indicators can be cogently captured by an examination of reasons for dying and reasons for living, to include asking the patient to rate the current intensity of motivation to die attached to each specific reason.

The six-item Brief Suicide Cognitions Scale [B-SCS (21)], for instance, can be translated into clinical interview questions across three domains important to recognizing the presence of identity-based hopelessness: unlovability, unbearability, and unsolvability. Although the formal scale includes only six items, clinicians can translate findings to interview questions that assess each domain:

- Do you ever feel completely unworthy of love or that there is nothing redeeming about you?
- Do you ever feel like your emotional pain is unbearable?
- Do you ever feel like your problems are unsolvable?

Perceived burdensomeness (23) can be similarly assessed with a simple question:

- Do you ever feel like a burden on your loved ones, or that they'd be better off if you were dead?

Unlike unlovability, unbearability, unsolvability and perceived burdensomeness, acquired capability to die is an observed variable that can be inferred from past behavior, prior trauma, abuse, and/or repeated exposure to death and violence:

- Have you ever done things to harm yourself, with no intention of dying, like cutting, burning, or hitting?
- As we've discussed, personal history is important to understanding how each of us experience the world around us. Have you had any experiences you would consider traumatic, particularly those involving exposure to violence or death?
- Some people experience events during the course of their work that might contribute to thoughts of suicide and the capacity to take their own life, such as exposure to violence and/or death. Has this been the case for you?

When factors suggesting the potential for elevated capability for suicide are identified, clinicians should integrate this information into their overall clinical assessment. Again, this is an observation that needs to be noted and factored into subsequent risk formulation. Elevated acquired capability suggests that suicidal episodes may have a lower threshold for activation, occur more often, potentially last for longer periods of time, and subsequent suicidal behaviors may be more lethal (23).

Firearm availability is another important element of elevated capability for suicide. Firearms are much more likely to be fatal as compared to other suicide attempt methods (24–30). Clinicians should therefore ask about firearm access, even with patients whose suicidal thinking involves other (non-firearm) methods. Additionally, clinicians should always inquire about access to multiple methods:

Even though you haven't mentioned a firearm, it's important to know if you own or have access to one. Do you own or have access to a firearm?

We have found that most individuals considering suicide think about more than one method. What other methods have you considered when having thoughts about killing yourself?

## Treatment Hesitancy and Non-Adherence With Clinical Care Recommendations

In a recent comprehensive review of randomized clinical trials targeting reductions in suicidal behavior, Rudd and Munoz-Perez (8) identified commonalties of treatments that work, with the recognition that assessing and responding to individual patient hesitancy and non-adherence with clinical care recommendations was a critical variable, along with the importance of having a clearly articulated adherence protocol. Non-adherence is a function of a broad range of variables ranging from straightforward barriers like a lack of transportation to more complex individual ones such as ambivalence about treatment, disruption created by active symptoms, and limited self-management skills necessary for full treatment engagement. Translating this finding into the clinical interview cuts across two variables. First, it is important for the clinician to recognize that patients who have made multiple suicide attempts often have reduced capacity for self-management and adherence due to limited self-regulation skills. Second, the potential for poor adherence can be assessed with a simple question every time the clinician makes a specific intervention request, such as using a crisis response or safety plan, following through with a means safety plan, practicing a newly learned skill, or taking their prescribed medication. This can be accomplished with a simple question following each request:

- It is not uncommon for people to feel uncomfortable about or struggle developing new skills. Accordingly, I'd like to better understand how you're feeling about doing the task we just discussed. How likely are you to do what we just discussed on a 1–10 scale, with 1 being you absolutely won't and 10 being you absolutely will?

This question not only can be integrated into the clinical interview, but also included as part of the overall adherence protocol within the treatment plan, because it allows the clinician an opportunity to proactively target, understand, anticipate, and respond to potential problems. In terms of the overall adherence protocol, the question also creates a unique window of opportunity for addressing low motivation and/or barriers to treatment engagement. Under these circumstances, clinicians can collaboratively engage patients in a conversation aimed at modifying or altering the recommended treatment strategy in a way that may increase motivation, adherence to clinical care recommendations, and eventual success in care. For example, the clinician might ask the following if a patient provides a low rating to the question above about likely strategy use:

- Your rating indicates it's likely that you won't be able to do what we just discussed. Can you help me better understand what might get in the way of doing the task?

Each identified barrier can then be discussed and targeted in a proactive fashion:

- What can we change about the task to increase the likelihood of you doing this? What steps can we take to move your rating to a 6 or above indicating it's more likely than not you'll be successful in completing the task? If needed, we can practice the skill or role play the strategy a few more times to increase your level of confidence before our time is up today.

## Assessing the Wish to Live and the Wish to Die

Finally, recent findings on the clinical utility of a patient's expressed wish to live and wish to die, coupled with reasons for living and reasons for dying (11–13), can be translated to a few simple questions that can be used routinely during assessment and ongoing treatment. More specifically, they can be assessed and tracked separately with simple self-ratings, providing additional insight into how the desire to die and desire to live are changing over time, and how these shifts correspond with the temporal dynamics of the individual's suicidal ideation:

- Can you rate your current wish to die on a scale of 1–10, 1 being no wish to die and 10 being a very strong wish to die? Let's talk about your reasons for dying. What are your reasons for dying? Why do you believe you need to kill yourself?
- Can you rate your current wish to live on a scale of 1–10, 1 being no wish to live and 10 being a very strong wish to live? Let's talk about your reasons for living. What are your reasons for living or for not killing yourself?

Identifying and discussing reasons for living also provides a useful platform for identifying and implementing interventions that can enhance cognitive flexibility and undermine the negative cognitive bias that characterizes suicidal states (12). Recent research further suggests that including a patient's reasons for living as a component of their crisis response or safety plan may lead to faster reductions in suicidal ideation, promote protective psychological states like hope and optimism, and support effective emotion regulation (31–33). As Brown et al. (34) noted, identifying a suicidal individuals' reasons for dying and reasons for living allows the clinician a mechanism to translate their ambivalence in concrete fashion, essentially a weighted value, coupled with the ability to actively engage their expressed ambivalence clinically, strategically intervene to move it in the direction of living (i.e., adding to the reasons for living list), and subsequently track it over the course of clinical care.

## Improving Risk Formulation

As mentioned at the outset, this article is not intended as a comprehensive approach to the suicide risk assessment clinical interview. Rather, the hope is threefold. First, to demonstrate that many recent advances can be translated in simple and efficient ways into the clinical interview to assess suicide risk. Second, that the questions can serve as a critical data source, supplementing information provided from standard assessment and screening tools, along with other resources. And third, that this approach provides an opportunity to humanize the assessment process, empowering the patient's voice, and help build a stronger therapeutic alliance essential to the successful provision of clinical care (17).

Of particular importance to the risk formulation process is the recognition and subsequent resolution of observed clinical discrepancies. These questions provide potentially critical self-report data to consider alongside standard screening and assessment tools. Data derived from these questions can be used strategically to explore potential areas of discrepancy between what a suicidal individual reports on an assessment tool and what they report during clinical interview. More important, though, than recognizing the discrepancy in reports, is allowing the suicidal individual the opportunity to reconcile and explain the discrepancy. Again, this can be accomplished with a few simple questions and geared specifically to the area of discrepancy:

- I noticed on one of the forms you completed earlier you endorsed an item indicating you had specific thoughts about how to kill yourself. Earlier you mentioned not having access to a method right now. I want to make sure we're on the same page and I accurately understand how you're doing. What did you do with the method you were thinking about when you completed the form? What other methods have you thought about?
- I noticed on one of the forms you completed earlier you mentioned having frequent thoughts about suicide. You reported not having thoughts of killing yourself now. Can you help me better understand how your suicidal thoughts come and go? Earlier we discussed the possibility of a pattern or cycling of your suicidal thoughts. Do you think that's what might be happening?

## Conclusion

As the volume of clinical research continues to grow, clinicians appropriately struggle with not only how best to keep pace with an ever-expanding field, but also how to interpret findings, integrate them into the risk assessment process, and do so in a manner that recognizes that clinical practice has realistic time constraints. The goal is to find ways to translate research advances into clinical practice in efficient and effective fashion. The suggestions offered above provide examples of how some of those scientific advances in suicide research can be integrated into day-to-day clinical practice in a simple, straightforward fashion.

## Data Availability Statement

The original contributions presented in the study are included in the article/supplementary material, further inquiries can be directed to the corresponding author/s.

## Author Contributions

MR and CB were involved in the conceptualization, development, and writing of the current manuscript. Both authors contributed to the article and approved the submitted version.

## Conflict of Interest

The authors declare that the research was conducted in the absence of any commercial or financial relationships that could be construed as a potential conflict of interest.

## Publisher's Note

All claims expressed in this article are solely those of the authors and do not necessarily represent those of their affiliated organizations, or those of the publisher, the editors and the reviewers. Any product that may be evaluated in this article, or claim that may be made by its manufacturer, is not guaranteed or endorsed by the publisher.
